# Injectable scaffold as minimally invasive technique for cartilage tissue engineering: in vitro and in vivo preliminary study

**DOI:** 10.1007/s40204-014-0031-x

**Published:** 2014-12-09

**Authors:** Atefeh Solouk, Hamid Mirzadeh, Saeed Amanpour

**Affiliations:** 1 Biomedical Engineering Faculty, Amirkabir University of Technology (Tehran Polytechnic), Tehran, Iran; 2 Polymer Engineering Faculty, Amirkabir University of Technology (Tehran Polytechnic), Tehran, Iran; 3 Cancer Research Center, Cancer Institute of Iran, Tehran University of Medical Sciences, Tehran, Iran

**Keywords:** Cartilage tissue engineering, Injectable scaffolds, poly (d,l-lactide-co-glycolide), Mesenchymal stem cells (MSCs)

## Abstract

Cartilage is a tissue with limited repair capacity and also sparse population of cells entrapped within a dense extracellular matrix, therefore, delivery of the cells to site of damaged cartilage can improve its healing potential. Synthetic biomaterials such as poly (d,l-lactide-co-glycolide) (PLGA) have been used as both preformed or injectable scaffolds in tissue engineering in order to carry and keep cells in the site of injury with minimal side effects. The injectable biocompatible polymeric scaffolds can reach to effected area via minimally invasive injection without need to open the joint, less painful approach and also having possibility to fill complicated shape defects. In this study, it was hypothesized that PLGA solved in *n*-methyl pyrrolidine (NMP) may act as a proper carrier for cell delivery to the site of the damage and also supports their growth. The results of in vitro assays including both live/dead (AO/PI) and MTT showed the majority of the cells were remained alive between 3 up to 21 days, respectively. The amount of resealed GAG from the mesenchymal stem cells (MSCs) which were in contact with both PLGA and alginate constructs (used as control) indicated that for day 7 MSCs in contact with alginate secreted more GAG (3.45 ± 0.453 µg/mL for alginate and 2.36 ± 0.422 µg/mL for PLGA matrices), but at longer times (21 days) cells in contact with PLGA elicited more GAG (6.26 ± 0.968 µg/mL for alginate and 8.47 ± 0.871 µg/mL for the PLGA matrices). Sol–gel systems comprising PLGA, NMP, and cells as well as alginate/cells were subcutaneously injected into four nude mice (each mouse had three injection sites). PLGA/NMP was solidify immediately and formed an interconnecting 3-D porous structure that allowed body fluid to penetrate through them. In vivo evaluation showed that PLGA/NMP scaffolds could support injected cells as a fibrocartilage tissue was formed after 6 months of injection. We found that PLGA/NMP system might be a proper minimally invasive therapeutics option for cartilage repair.

## Introduction

There are no nerves or blood vessels in cartilage tissue, and problematic point is that when it damages due to any reason such as disease or trauma, it does not heal spontaneously and also leads to sever pain and disability (Capito et al. 2005; Kreuz et al. 2013). Although numerous treatment protocols are currently employed clinically, few approaches, if any, exist which are capable of consistently restoring long-term function to damaged articular cartilage and rest are failed of completely restore cartilage structure and function because of invasiveness of the methods and complex properties of the cartilage tissue (Vinatier and Mrugala 2009).

Since, cartilage is a tissue, with sparse population of cells entrapped within a dense extracellular matrix (ECM), therefore, delivery of cells to site of damaged cartilage using a carrier may improve its healing potential (Puppi and Chiellini 2010). Therefore, tissue engineering approaches using scaffold architecture for delivery of cells in an organized manner to the site of cartilage defects, offer great promise as repair strategies (Lara-Curzio and Readey 2004). In overall, the tissue engineering scaffolds can be divided into two main types including preformed and injectable (Zilberman 2011; Parka et al. 2013). From clinical perspective, the use of injectable scaffolding materials for in vivo tissue regeneration is attractive because it allows cell implantation through minimally invasive and routine surgical procedures (Bakhshi and Vasheghani-Farahani 2006; Francisco et al. 2013). In fact, this approach is less invasive and less painful compared to opening the joint and implants combination of cells and scaffold in it. Another advantage of using injectable scaffolds is that they can easily fill defects of various sizes and shapes without any need to fabricate scaffolds of complicated shapes (Hu et al. 2008). Injectable, in situ forming materials have been extensively used as career in drug delivery systems (DDS). As it was mentioned in a review published by Mikos et al., due to the advantages of injectable materials for both drug delivery systems and tissue engineering, the experience transfer from injectable carrier in field of DDS in order to reach appropriate in situ forming scaffolds is warranted (Jia and Kiick 2009). The application of injectable implant system comprises a water-insoluble biodegradable polymer, poly (d,l-lactide-co-glycolide) (PLGA) a copolymer with a 50:50 molar ratio containing carboxyl end groups, dissolved in a water-miscible and physiologically compatible organic solvent, *N*-methyl-2-pyrrolidone (NMP) already has been investigated for DDS applications (Bakhshi and Vasheghani-Farahani 2006; Tahereh Darestani Farahani et al. 2005; Astaneh et al. 2009). Considering the necessary characteristics for tissue engineering scaffolds and how current injectable systems which were used as drug carrier could be modified to facilitate their use as injectable scaffolds was the motivation of current study. In fact, in this research, the potential of PLGA/NMP injectable system to act as cell delivery carrier was investigated. Upon injection into an aqueous environment, the organic solvent diffuses into the surrounding environment while water diffuses into the polymer matrix. Then, the polymer precipitates in contact with water and results in a solid polymeric implant and formation of an interconnecting 3-D porous structure that allowed body fluid to penetrate through it.

The rationale behind of this work was a hypothesis that might be simultaneous injection of cells and scaffold create a solidified microenvironment to motivate cell growth and proliferation. To compare the biological function of PLGA/NMP, alginate, a biocompatible hydrogel which has been shown to be effective cell delivery carrier (Marijnissen et al. 2002; Sah et al. 2003; Tiğli and Gümüşderelioğlu 2009), was also used.

## Materials and methods

### Scaffold preparation

#### Sample 1: based on PLGA

Poly (d,l-lactide-co-glycolide) (PLGA) 50:50 (RG 505, inherent viscosity = 0.54 dL/g in chloroform at 25 °C, Mw = 24,000) to a biocompatible solvent, *N*-methyl-2-pyrrolidone (NMP, Merck, Germany) in solution between 30 % of PLGA and 70 % of NMP, both components are FDA approved, was prepared. It was seen that 70:30 w/w PLGA: NMP solution had enough low viscosity for easy injection. The PLGA solutions were gamma irradiated (dose of 25 kGy). Then, injected into a cylindrical mould with height twice than width that was filled with phosphate-buffered saline (PBS), exchanging the NMP and water the porous scaffold was prepared.

#### Sample 2: based on alginate

Alginate solution of 1.2 % was prepared by adding alginic acid (Fluka, Biochemica) to 0.9 % NaCl while stirring. Afterward the solution of alginic acid in saline was injected into a tube containing calcium chloride solution through a syringe. The alginate droplets crosslinked and resulted in alginate beads.

### Scanning electron micrograph of scaffold

Surfaces and cross-sections microstructure of the injected PLGA/NMP system into PBS solution were studied using a scanning electron microscope (SEM), Vega-II XMU (Tescan USA Inc). For this purpose, the injected substrate were remained in PBS for duration of 48 h and broken in liquid nitrogen after freezing (Astaneh et al. 2009).

### Mechanical property of the scaffolds

First, the hydrogels comprising PLGA/NMP without cells were injected into a vital containing aqueous solution to be solidified as a scaffold. Thereafter, the compression modulus of the scaffolds was evaluated using ASTM standard procedure (ASTM 1996). These tests were performed utilizing a dynamic servo hydraulic testing machine (HCT 25-400, Zwick/Roell, Germany). Data were analyzed using ToolKit98 (Zwick/Roell, Germany) software. Three cylindrical samples with height twice than of their width were prepared and compressed to their half height at a speed of 0.5 mm/min. At this point the maximum load was measured and data expressed.

### In vitro assays

#### Extraction of the samples

In order to evaluate the effect of PLGA-based and alginate-based scaffolds on the cell growth and proliferation, an extraction process was done according to the ISO 10993-5. The biopsy of each scaffold with the weigh within a range of 0.1–0.2 g was taken and 1 mL of culture medium was added to each ones. After 7 and 21 days these mediums were taken out to use in cell proliferation assay. A specified amount of culture medium was kept in the same condition as a negative control.

#### Cell proliferation assay

Both human bone marrow-derived mesenchymal stem cells (MSCs) and mouse chondrocyte cells which were used in this study were kindly provided by Royan Institute (Tehran, Iran). Proliferation rate of MSCs on the samples were measured using MTT assay. Briefly, at the first day MSCs were plated into a 96-well microtiter plate at 1 × 10^4^ cells/well. After 24 h, the culture medium of each well was removed and replaced with 90 μL extract plus 10 μL FBS. In the next 24 h, the medium eliminated and 100 μL of a 0.5 mg/mL solution of MTT (Sigma, USA) was added to each well followed by incubation for 5 h at 37 °C. The purple formazan crystals (formed in the mitochondria of the cells) were detected and later dissolved by addition of 100 μL isopropanol (Sigma, USA) per well. The plates were then incubated at 37 °C for 15 min prior to absorbance measurements. The optical density (OD) was recorded on a multi well microplate reader (ICN, Switzerland) at 545 nm and normalized to the control OD.

#### Live/dead assay

About 1 × 10^4^ MSC cells were mixed with 1 mL of alginate solution or poured onto PLGA scaffolds, then placed into a 12-well cell culture plate, and incubated at 37 °C for 3 days (*n* = 3). Cell viability in the alginate hydrogel was assessed by using acridine orange-propidium iodide (AO/PI) staining. Briefly, the stock solution (AO: 670 mmol/L, PI: 750 mmol/L) was prepared with Dulbeccos solution and kept in the dark at 4 °C. Just before use, 0.01 mL AO and 1.0 mL PI were mixed, diluted by 10 times with Dulbeccos solution, and then passed through a 0.22-µm filter membrane. The scaffolds containing MSC were incubated with the AO/PI mixture and observed under a fluorescence microscope later to evaluate the amount of live cells which stain in green (AO) and also dead cells which will be colored in red (PI) (Hao and Wen 2010).

#### Proteoglycan content analysis

GAG content was determined using DMMB assay for the MSCs seeded on each PLGA and alginate scaffolds for duration of 7 and 21 days. Both samples were papain digested and analyzed for glycosaminoglycan (GAG) content using the DMMB-dye binding assay. Briefly, 50 µl of papain digested sample was incubated with 2 mL of DMMB-dye, and the reaction was observed using an ELISA plate reader at 545 nm, with chondroitin sulfate (shark cartilage extract, Sigma) used as a standard (Park et al. 2007).

### In vivo assay

**Fig. 1:**
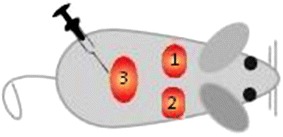
Three injection sites for each nude mouse, *site 1* PLGA/NMP and cells, *site 2* alginate gel and cells, and *site 3* control (i.e., only cells)

A double syringe comprising the two separate cylinders and 25-gauge needle was used in order to deliver adequate amount of cells and injectable scaffolds into subcutaneous space of four nude mice simultaneously as presented in Fig. 1. In fact, one syringe contained either 70:30 v/v of PLGA: NMP solution or alginate/CaSO_4_ mixture (which had previously been sterilized by a 0.22 μm filter) and another one was contained the mouse chondrocyte cell with the density of 1 million cells/mL. The amount of cells and polymer solutions in each injection site are presented at Table 1. Mice were sacrificed at 2 and 24 weeks by anesthesia overdose and samples were harvested from cell alone and cell-polymer samples. Cartilage structures, if any, were excised, fixed in 10 % buffered formalin for pathological investigation. Since a nude mouse has tiny body then a subcutaneous space of it also has low capacity. Therefore, for cover three injection sites for any mouse, each injection contained 0.3 mL combination of cells and polymer solution.

#### Histology

Samples from each injection site and time point were rinsed in 2.5 mL PBS for 1 h and then fixed in 10 % neutral-buffered formalin (Sigma-Aldrich). After fixation, these samples were dehydrated by immersion in a series of ethanol solutions (70, 80, 85, 90, 95, and 100 %) and xylene solutions in ethanol (50 and 100 %). Specimens were then embedded in paraffin and cross-sectioned to a thickness of 20 µm using a microtome (Microm, Walldorf, Germany). Sections from all groups were simultaneously stained with hematoxylin and eosin (Park et al. 2005). After fixation with 10 % phosphate-buffered formalin for at least 24 h, specimens were embedded within paraffin and sectioned. Using standard histochemical techniques, serial sections were stained with hematoxylin and eosin stains (Chang et al. 2001).

### Statistical analysis

Experiments were run in triplicate for each sample. All data are expressed as mean ± standard deviation (SD). Utilizing one-way analysis of variance (ANOVA) SPSS 16.0 software which followed by Tukeys HSD post hoc test, statistical analysis between groups was performed. *P* values of less than 0.05 and less than 0.001 were considered significant and very significant, respectively.

## Results and discussion

### Scaffold characterization

#### Scanning electron microscopy

**Fig. 2:**
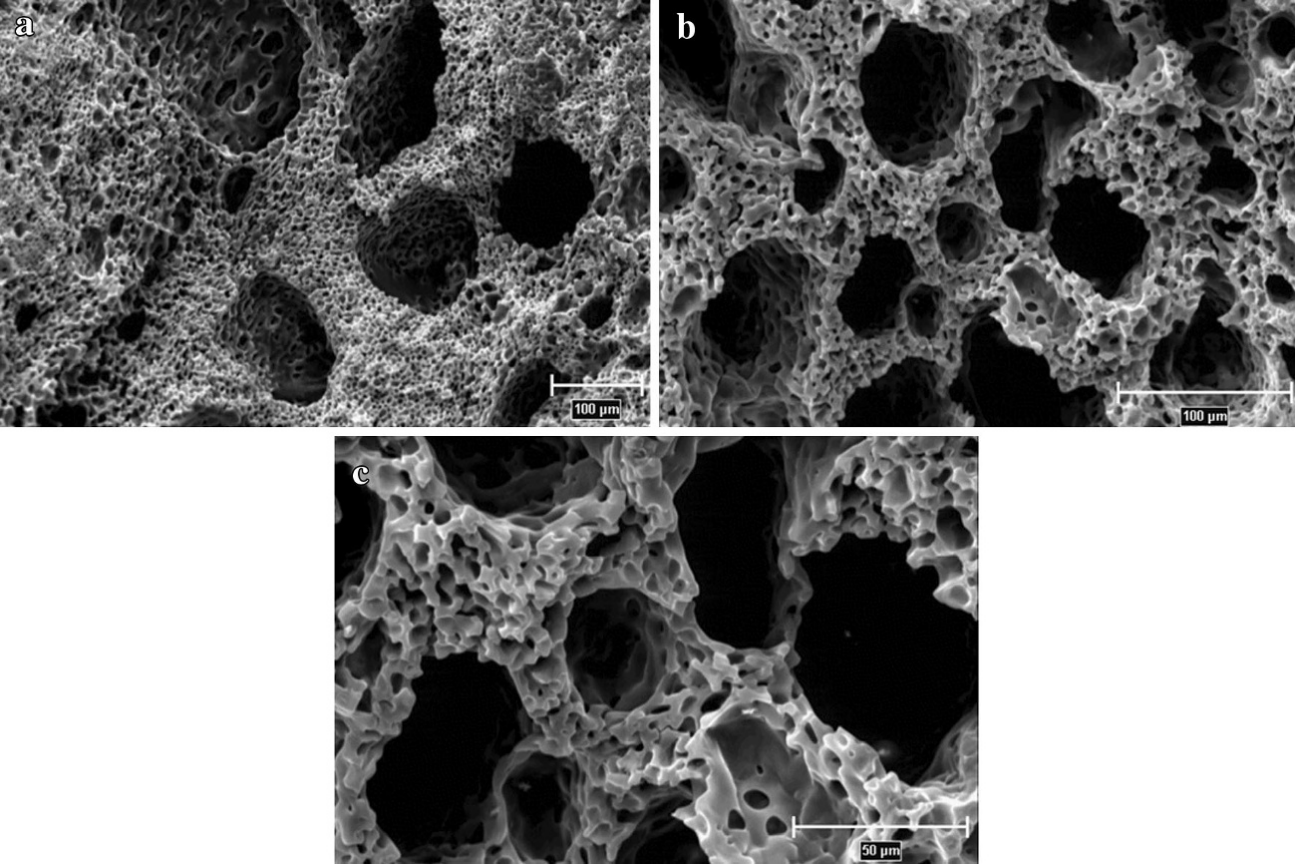
Morphological observations by SEM, PLGA scaffold **a** (×125), **b** (×250) and **c** (×500)

Figure 2 shows the surface micrographs of three solidified PLGA/NMP scaffolds 3 days after their injection into an aqueous media with different magnifications (125×, 250 and 500). It is known that the pore size of the scaffold plays an important role in cell binding, migration, and ingrowth. Although nutrient materials, gases, and metabolic waste can be transported more easily via interconnected large pores in the scaffold, large pores can lead to low cell attachment and intracellular signaling. In contrast, small pores can have the opposite effect, in which cell attachment is promoted, but there is poor nutrient and gas delivery (Annabi et al. 2011). The broad range for pore sizes with good distribution of small and big pore diameters can be seen in SEM pictures of Fig. 2. The average diameter of large pores was nearly about 129.44 ± 23 µm. This anisotropic distribution of pores is favored for cartilage tissue engineering as mentioned in the literature (Annabi et al. 2011).

#### Mechanical property of the scaffolds

The scaffolds which are designed for load-bearing applications such as musculoskeletal tissues should provide sufficient mechanical support to match the mechanical property of the host tissue to bear the in vivo stresses and loadings. In the other words, mechanical compatibility or similarity (matching) between the scaffold and tissue plays a crucial role in homeostasis, remodeling, and repair of load-bearing tissues, such as bone and cartilage (Duncan and Turner 1995; Carter et al. 1988; Jin et al. 2003). Ideally, the most desirable mechanical properties for a scaffold are those closest to real tissue. The compression tests carried out in this project were used to evaluate the mechanical properties of PLGA injectable scaffold. As it is reported in the literature (Southgate et al. 2009), natural human cartilage has compression modulus in range of 0.5–1.5 (MPa) (Yuehuei and Kylie Martin 2010). The related data for PLGA scaffold without cells shows compression modulus of 0.5 ± 0.06 MPa which is near to minimum amount of aforementioned range. It seems likely that ECM secretion via chondrocyte cells could also improve the scaffolds mechanical properties. However, these data are not available now.

**Table 1:** Content of cells and polymer solutions in each injection site

Site of injection	Content				
	Volume of injected cells (mL)	Number of injected cells (×10^6^)	Volume of PLGA in NMP (mL)	Volume of alginate in NaCl solution (mL)	Total volume of injection (cells + polymer solution) (mL)
1	0.15	0.6	0.15	–	0.3
2	0.15	0.6	–	0.15	0.3
3	0.3	0.6	–	–	0.3

### In vitro assays

#### MTT assay

**Fig. 3:**
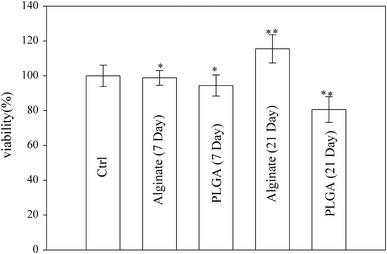
The viability of MSCs after exposed to 7 and 21 days (*n* = 3, mean ± SD), values of *P* > 0.05 were considered no significant (*) and *P* < 0.05 were considered significant (**). (*C* is stand for crosslinked samples)

After 3 days of cell culture, the cell proliferation was determined by the MTT method. The MTT is a reliable assay method for measuring cell viability in different substrates, especially in rigid and porous scaffolds. This assay determines viable cell numbers and is based on the mitochondrial conversion of the tetrazolium salt, 3(4, 5-dimethylthiazol-2-yl)-2, 5-diphenyltetrazoliumbromide (MTT) (Karbasi et al.^2005^; Park et al. 2005). The MTT assay was performed at 7 and 21 days to determine cell growth within both PLGA and alginate scaffolds and results are presented in Fig. 3. Cell proliferation remained steady in both samples after 7 days, while a considerable increase in cell amount could be seen at day 21 of alginate sample. This significant increase at day 21 can be attributed to the difference in nature of PLGA and alginate scaffold. It has been mentioned in the literature that synthetic material such as PLGA have less cell adhesion and growth in comparison with naturally derived polymers such as alginate (Chang et al. 2001). Also, it is reported that PLGA due to enzymatic degradation converts to lactide and glycolide acids which led to decreasing of physiologic pH surrounding tissues and subsequently prevent cell growth (Sung et al. 2004).

#### Live/dead assay

**Fig. 4:**
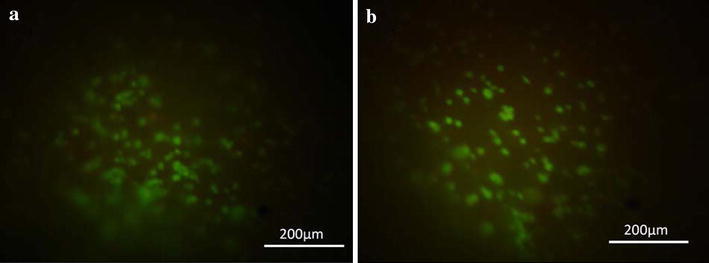
The AO/PI staining of **a** alginate, **b** PLGA, MSCs remained >90 % viable in both scaffold cultured after 7 day, *AO* green, *PI* red

To visualize the cell viability in the scaffolds, living and dead cells in PLGA and alginate matrix were fluorescently stained using AO/PI staining. As shown in Fig. 4, the MSC cells remained viable in both PLGA and alginate matrix after being cultured for 3 day in vitro to some extent. Comparison the number of living cells (stained green) and dead cells (stained red) in PLGA and alginate substrate is shown in Fig. 4a, b, respectively, it looks like that nearly no dead cells is seen in alginate matrix meanwhile the number of red points is more in Fig. 4b. This result is consistent with the MTT assay outcome which shows better cell viability for alginate rather than PLGA in close system of in vitro (Hao and Wen 2010; Ibusuki et al. 2003).

#### Proteoglycan content analysis

**Fig. 5:**
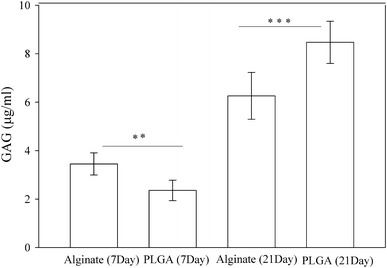
Total GAG contents in scaffolds after 7 and 21 days. (*n* = 3, mean ± SD), values of *P* < 0.05 were considered significant (**) and *P* < 0.001 were considered very significant (***)

The amount of resealed GAG from the cells which were in contact with PLGA and alginate constructs for duration of 7 and 21 days is shown in Fig. 5. The total GAG at day 7 was 3.45 ± 0.453 µg/mL for alginate and 2.36 ± 0.422 µg/mL for the PLGA matrixes, and the total GAG at day 21 was increased to 6.26 ± 0.968 µg/mL for alginate and 8.47 ± 0.871 µg/mL for the PLGA matrices. At day 7, alginate construct encapsulating MSC cells possesses significantly higher GAG content in comparison with PLGA. The rationale behind this variation might be due to alginate nature or hydrophobicity of PLGA which limited cell adhesion on PLGA as shown the same result in MTT assay.

For both scaffolds, an increase in GAG production was observed with longer culture time period since 7–21 days. At last, PLGA career showed more cell supporting and higher GAG content in comparison with alginate scaffolds (Park et al. 2005). This observation for day 21 is in contradiction with the results for day 7. This contradiction might be due to the weakness in mechanical properties of alginate which make it very loose to support cell activity and ECM secretion meanwhile PLGA matrix (with higher mechanical properties) can be better support for cells in the longer times (Dai et al. 2010; Mercier et al. 2004).

### In vivo assay

**Fig. 6:**
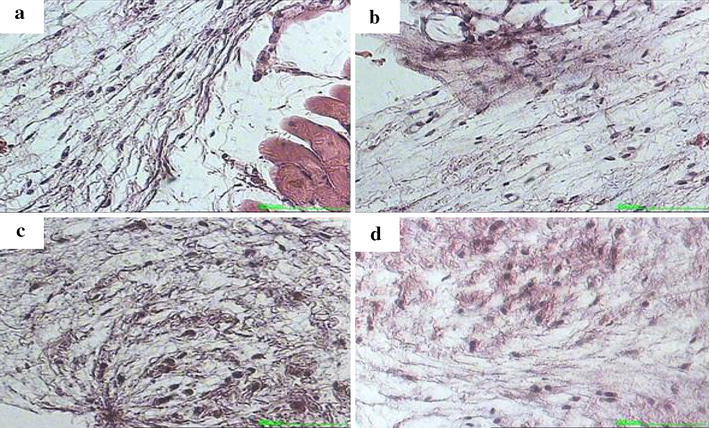
Light micrographs of a histological slide after 2 weeks of injection, **a** site 2 (cell + alginate) and **b** site 1 (cell + PLGA), *scale bar* 250 μm

**Fig. 7:**
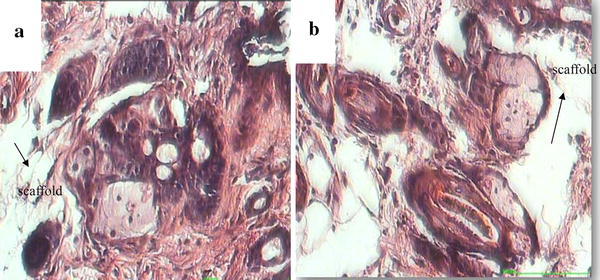
Light micrographs of a histological slide after 24 weeks of injection, **a**, **b** site 2 (cell + alginate) and **c,d** site 1 (cell + PLGA), *scale bar* 250 μm

Histological analysis by H&E staining of combination of cells and scaffolds retrieved at 2 and 24 weeks is shown at Figs. 6 and 7, respectively. The in vivo results after 2 weeks showed that in site 1 (PLGA/NMP) and 2 (alginate), there were chondrocyte cells which remained alive but no tissue samples could be harvested from cell-injected site without scaffold (site 3) because there was no tissue formation over there as a result of cells migration, apoptosis, or reabsorption (Shafiee and Soleimani 2011). Our investigations after 24 weeks showed that chondrocyte accompanied by PLGA polymer regenerated more mature and well-formed cartilage, as evidenced by bigger chondrocyte core size in comparison with alginate (Mercier et al. 2005). In this study, a natural polymer was used as control because it is known that naturally occurring biopolymers such as alginate due to their biodegradability, low toxicity, and low disposal costs are proper choice to act as tissue engineering scaffold. Furthermore, alginate is especially an appropriate biomaterial for the cartilage tissue as it can closely mimics the natural environment or cartilage ECM (Fan et al. 2006). H&E staining results after 24 weeks of in vivo implantation showed comparable results with outcome of proteoglycan production amount in vitro (mentioned in Sect. “Proteoglycan content analysis”). Actually, the size of cell’s core in site 1 (PLGA) was bigger than site 2 (alginate), that might be due to better support to cells behalf stiff PLGA matrix rather than loose alginate carrier which may causes the cell shrinkage and apoptosis. The fact that obtained in vitro and in vivo results of PLGA scaffolds is comparable to those of alginate makes a support for using this synthetic injectable structure for repairing damaged cartilage. Histopathological investigation showed that there is no significant difference between the chondrocyte core size which was accompanied by either PLGA or alginate.

## Conclusion

In many clinical situations involving replacement of hard or soft tissue, the aims of minimizing the need for invasive surgery, avoiding the medical complications associated with harvested tissue, and overcoming the limitations of preformed scaffolds have assumed primary importance. Therefore, the use of noninvasive, injectable biomaterials with the capacity to fill irregular defects is so attractive and responds to these concerns. When properly designed, an injectable scaffold can provide a structure that encapsulates a homogeneous distribution of cells and bioactive molecules that stimulate the regeneration of bone and cartilage in a biomimetic fashion (Hu et al. 2008; Mercier et al. 2005; Migliaresi et al. 2007). In this study, the potential of a biocompatible system including PLGA and NMP which both could gained FDA approval for human use was assayed in order to act as cartilage scaffold, and the in vitro and in vivo results showed promising results. The MTT results showed that although MSCs cells had better growth and viability in contact with alginate gel rather than PLGA scaffolds, but the amount of produced extracellular matrices (e.g., glycosaminoglycan) of them was better in contact with PLGA which had higher mechanical support for the cells. The positive effect of mechanical stimulation on GAG expression during the differentiation of MSCs into chondrocytes was observed in our previous study too (Karkhaneh et al. 2014). The in vivo results implied that as the PLGA scaffold degrades, the porous spaces could be replaced with regenerating fibro-cartilage tissue. Direct injection of chondrocyte suspensions without any scaffold was conducted as a control experiment, but the localization of transplanted chondrocytes was difficult to control, and new cartilage tissue formation was not observed. We found that PLGA/NMP system might be a proper minimally invasive therapeutics option for cartilage regeneration.
