# Erratum to: Injectable scaffold as minimally invasive technique for cartilage tissue engineering: in vitro and in vivo preliminary study

**DOI:** 10.1007/s40204-015-0036-0

**Published:** 2015-02-24

**Authors:** Atefeh Solouk, Hamid Mirzadeh, Saeed Amanpour

**Affiliations:** 1 Biomedical Engineering Faculty, Amirkabir University of Technology (Tehran Polytechnic), Tehran, Iran; 2 Polymer Engineering Faculty, Amirkabir University of Technology (Tehran Polytechnic), Tehran, Iran; 3 Cancer Research Center, Cancer Institute of Iran, Tehran University of Medical Sciences, Tehran, Iran

## Erratum to: Prog Biomater (2014) 3:143–151 DOI 10.1007/s40204-014-0031-x

Unfortunately, the presentation of Fig. 4 was incorrect.

The corrected Fig. 4 is given below.

**Fig. 4:**
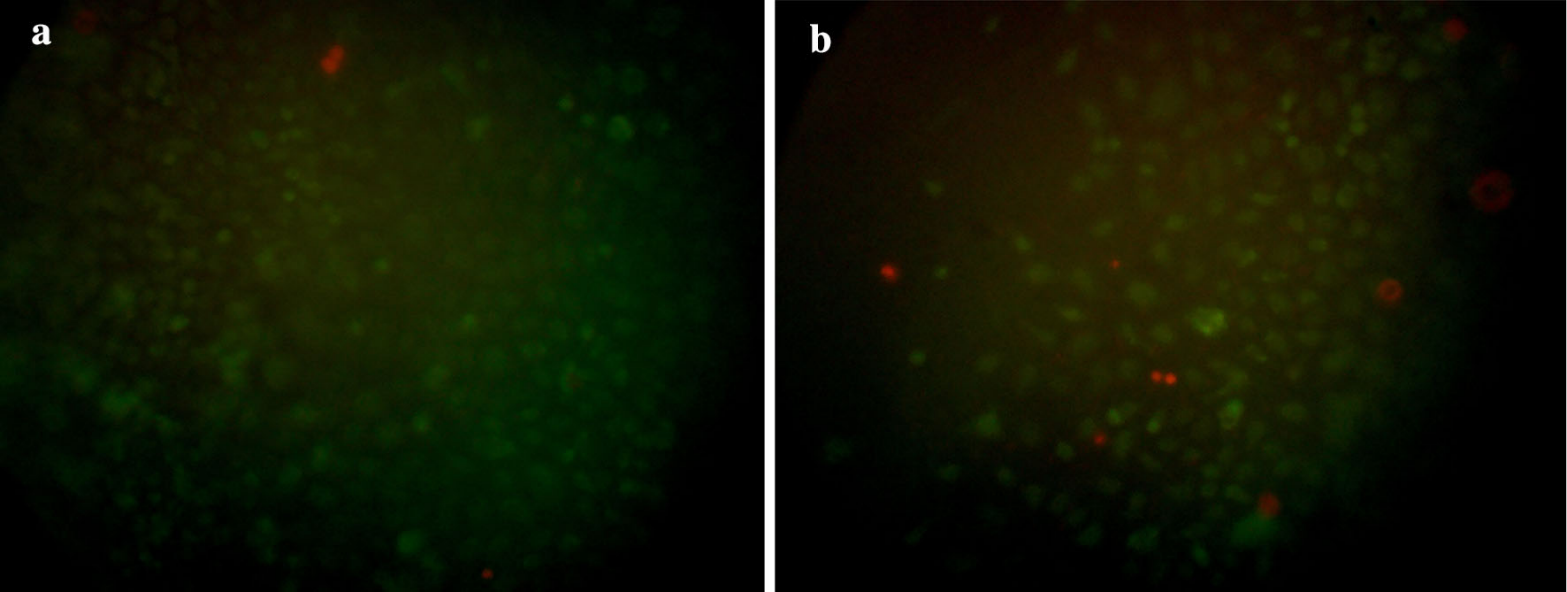
The AO/PI staining of **a** alginate, **b** PLGA, MSCs remained >90 % viable in both scaffold cultured after 7 day, *AO* green, *PI* red

